# Egr-1 mediates the suppressive effect of IL-1 on PPARγ expression in human OA chondrocytes

**DOI:** 10.1186/1479-5876-10-S3-P39

**Published:** 2012-11-28

**Authors:** Salwa Sarah Nebbaki, Fatima Ezzahra El Mansouri, Nadia Zayed, Mohamed Benderdour, Johanne Martel-Pelletier, Jean-Pierre Pelletier, Hassan Fahmi

**Affiliations:** 1 Osteoarthritis Research Unit, University of Montreal Hospital Research Center (CRCHUM), Notre-Dame Hospital, Montreal, Quebec, Canada; 2 Centre de recherche Hôpital Sacré-Coeur de Montréal, Montreal, Quebec, Canada

## Background

Peroxisome proliferator-activated receptor gamma (PPARγ) is a ligand activated transcription factor and member the nuclear hormone receptor superfamily. Several lines of evidence indicate that PPARγ have protective effects in osteoarthritis (OA). Indeed, PPARγ has been shown to down-regulate several inflammatory and catabolic responses in articular cartilage and chondrocytes and to be protective in animal models of OA.

## Aim

We have previously shown that IL-1 down-regulated PPARγ expression in OA chondrocytes. In the present study we will investigate the mechanisms underlying this effect of IL-1.

## Methods

Chondrocytes were stimulated with IL-1, and the level of PPARγ and Egr-1 protein and mRNA were evaluated using Western blotting and real-time reverse-transcription polymerase chain reaction, respectively. The PPARγ promoter activity was analyzed in transient transfection experiments. Egr-1 recruitment to the PPARγ promoter was evaluated using chromatin immunoprecipitation (ChIP) assays. Small interfering RNA (siRNA) approaches were used to silence Egr-1 expression.

## Results

We demonstrated that the suppressive effect of IL-1 on PPARγ expression requires de novo protein synthesis and was concomitant with the induction of the transcription factor Egr-1. ChIP analyses revealed that IL-1 induced Egr-1 recruitment at the PPARγ promoter. IL-1 inhibited the activity of PPARγ promoter and overexpression of Egr-1 potentiated the inhibitory effect of IL-1, suggesting that Egr-1 may mediate the suppressive effect of IL-1. Finally, Egr-1 silencing with small interfering RNA blocked IL-1-mediated down-regulation of PPARγ expression.

## Conclusion

These results indicate that Egr-1 contributes to IL-1-mediated down-regulation of PPARγ expression in OA chondrocytes and suggest that this pathway could be a potential target for pharmacologic intervention in the treatment of OA and possibly other arthritic diseases.

